# Self-Reported COVID-19 Vaccines’ Side Effects among Patients Treated with Biological Therapies in Saudi Arabia: A Multicenter Cross-Sectional Study

**DOI:** 10.3390/vaccines10060977

**Published:** 2022-06-20

**Authors:** Lama T AlMutairi, Wesal Y Alalayet, Sondus I Ata, Khalidah A Alenzi, Yazed AlRuthia

**Affiliations:** 1 Department of Pharmacy, King Khalid University Hospital, Riyadh 12372, Saudi Arabia; lamaturki.11@gmail.com (L.T.A.); walalayet@ksu.edu.sa (W.Y.A.); sata@ksu.edu.sa (S.I.A.); 2 Regional Drug Information and Pharmacovigilance Center, Ministry of Health, Tabuk 47913, Saudi Arabia; ph_kh@hotmail.com; 3 Department of Clinical Pharmacy, College of Pharmacy, King Saud University, Riyadh 11451, Saudi Arabia; 4 Pharmacoeconomics Research Unit, Department of Clinical Pharmacy, College of Pharmacy, King Saud University, Riyadh 11451, Saudi Arabia

**Keywords:** COVID-19, Oxford–AstraZeneca COVID-19 Vaccine, Pfizer COVID-19 vaccine, biological products, monoclonal, antibodies, Saudi Arabia

## Abstract

Objective: The aim of this study was to explore the side effects of COVID-19 vaccines among a mixed gender sample of patients on monoclonal antibody biologics (mAbs) in Saudi Arabia. Methods: This was a prospective questionnaire-based cross-sectional study in which adult patients (≥18 years) on mAbs who had received at least one dose of COVID-19 vaccine from three tertiary care centers in Saudi Arabia were included. Descriptive statistics and univariate logistic regressions were conducted to present the vaccine side effects and examine the association between the reported side effects and vaccine type. Results: Four-hundred and seventeen patients, with a mean age of 39 years, consented to participate. Approximately 82% and 18% of the participants received Pfizer–BioNTech and Oxford–AstraZeneca vaccines, respectively, and nearly 71% received two doses of the vaccine. Diarrhea (9.59%), fever (51.32%), headache (32.13%), hypotension (13.67%), palpitation (9.11%), and temporary loss of smell (5.28%) were the most commonly reported side effects. Conclusion: COVID-19 vaccines are generally safe for patients treated with mAbs. Future studies should examine the rates of side effects across different COVID-19 vaccines among patients on mAbs using more robust study designs and representative samples.

## 1. Introduction

The Coronavirus Disease-2019 (COVID-19) first emerged in Wuhan, Hubei, China, in late 2019 and rapidly spread thereafter to many countries. It was declared a pandemic by the World Health Organization (WHO) on 11 March 2020 [1]. COVID-19 infection presentations range from asymptomatic or mild symptoms to respiratory failure and death [2]. As of 4 March 2022, more than 440 million COVID-19 cases and 5.5 million deaths had been reported based on the WHO COVID-19 dashboard [3]. In Saudi Arabia, there had been more than 747,000 confirmed COVID-19 cases and 9000 deaths as of 4 March 2022 [4]. Therefore, the Saudi government, like most of the world’s governments, raced to contain this pandemic through different measures, such as social distancing, temporary lockdowns, travel bans, and most importantly, vaccination [5]. More than 61 million doses of COVID-19 vaccines have been administered to the Saudi public in the ongoing mass vaccination campaigns [6]. Currently, there are several COVID-19 vaccines that have been approved by different regulatory bodies, such as the United States Food and Drug Administration (USFDA), under the emergency-use authorization [7]. These vaccines can be either RNA–based, such as Pfizer–BioNtech (BNT16b2) and Moderna (mRNA-1273) [8], non-replicating viral vectors, such as Oxford–AstraZeneca (AZD1222) [9], protein-based, such as Novavax (NVX-CoV2373) [10], and inactivated virus, such as Sinovac Biotech (CoronaVac) [11]. Although the efficacy rates of COVID-19 vaccines are variable, they have overall proven to be effective in reducing the rates of hospitalization and mortality [12]. Nonetheless, the efficacy of COVID-19 vaccines was significantly lower among immunocompromised patients, such as patients with cancers, rheumatoid arthritis, and multiple sclerosis, in comparison to their immunocompetent counterparts [13].

According to the Centers for Disease Control and Prevention (CDC) and the WHO, the common side effects of COVID-19 vaccines include fever, fatigue, headaches, chills, nausea, and injection site reactions [14]. However, some rare but serious adverse events, such as anaphylaxis, venous thromboembolic events, cerebral venous thrombosis, myocarditis or myopericarditis, Guillain–Barré syndrome, and bleeding were observed [14,15,16,17,18]. Nevertheless, the most frequently reported side effects of COVID-19 vaccines were chills, fatigue, nausea, vomiting, flu-like symptoms, palpitation, eye pain, malaise, pyrexia, peripheral swelling, injection site pain, chest pain, decreased appetite, arthralgia, myalgia, headache, dizziness, tremor, dyspnea, rash, oropharyngeal discomfort, hyperhidrosis, diarrhea, abdominal pain, and cough [19,20,21,22].

Patients treated with immunomodulators or disease-modifying therapies, such as in the case of multiple sclerosis, inflammatory bowel disease (IBD), and rheumatoid arthritis (RA) are at an increased risk of infections and impaired vaccine response [13,23,24,25]. Most of the studies that assessed the impact of vaccines on immunosuppressants have only examined the humoral response to vaccines without exploring their cellular response [26]. For rheumatoid arthritis patients, the humoral response after receiving the influenza vaccine was significantly increased among patients treated with methotrexate; however, this was not seen among rheumatoid arthritis patients treated with rituximab [27]. Moreover, the use of methotrexate with tocilizumab or tofacitinib was associated with diminished responses to influenza and pneumococcal vaccines according to a meta-analysis that included nine studies that evaluated the effect of antirheumatic drugs on influenza and pneumococcal vaccine immunogenicity [24]. However, the cellular immunity to influenza vaccination in rheumatoid arthritis patients treated with rituximab or other disease-modifying antirheumatic therapies (DMARDs) was maintained despite a reduced humoral response among patients treated with rituximab [28]. Therefore, many medical societies and experts recommend vaccinating patients with autoimmune disorders despite their reduced humoral responses to influenza, pneumococcal, and COVID-19 vaccines [27,29,30,31,32].

Although multiple studies have explored commonly reported adverse effects of COVID-19 vaccines among the general population [14,19,20,21,33], few studies have examined the reported side effects among patients with autoimmune disorders on biological therapies [34]. Therefore, we aimed to explore COVID-19 vaccine side effects among patients treated with biological therapies for different medical disorders and compare the rates of these side effects across COVID-19 vaccines in Saudi Arabia.

## 2. Methods

### 2.1. Design and Settings

This was a prospective questionnaire-based, cross-sectional study that was conducted between 2 August and 1 December 2021 in three tertiary care hospitals in the cities of Riyadh and Tabuk, Saudi Arabia. Adult patients (≥18 years) treated with monoclonal antibodies (mAbs) for different medical conditions, such as rheumatoid arthritis and Crohn’s disease, and who, according to their medical records, had received at least one dose of COVID-19 vaccine within the past 2 months were included in the study. Patients with cancer, pregnant women, patients under 18 years of age, and those who were treated with mAbs for less than 12 months were excluded. The vaccines (Pfizer–BioNTech, Oxford–AstraZeneca, Moderna, etc.) were administered to patients based on their availability, and there was no specified type of vaccine that should be administered to patients on mAbs.

### 2.2. Questionnaire and Data Collection

In order to explore the side effects of COVID-19 vaccines, a 26-item questionnaire was developed (Appendix A). The questionnaire inquired about the sociodemographic characteristics of the patients (gender, age, educational level, marital status, and employment status), medical characteristics (body mass index, the main medical condition that mAb was prescribed for, comorbid medical conditions, and prescription medications), smoking status, biologic product name and its first and last dates of administration, and details of COVID-19 vaccination (date of vaccination, vaccine name, and checklist of different reported vaccine side effects). The included vaccine side effects were retrieved from multiple studies that explored COVID-19 vaccine side effects [14,19,20,21,33]. The questionnaire was forward translated to Arabic by the first author, and was then reviewed by the rest of the research team since it only included commonly used terms in practice that could be easily translated. Furthermore, the questionnaire was backward translated by another pharmacist whose native language is English and who is proficient in Arabic. No significant differences were observed, and the face and content validity of the questionnaire were checked by two researchers in health outcomes. To ensure the comprehensibility of the questionnaire’s items, the first 25 interviewed participants were regarded as a pilot sample. No significant changes were made since the questionnaire’s items were clear to all interviewed participants. Patients with autoimmune diseases on mAbs are usually treated in ambulatory care clinics where they receive their mAbs for their respective illnesses. Therefore, three pharmacists who followed a structured interview protocol approached the patients who fit the inclusion criteria during their scheduled appointments in the ambulatory care clinics in the three hospitals and presented them with a written consent form that explained the objective of the study and the right of the patients to withdraw at anytime during the interview. Those who consented to participate were interviewed for 10 to 15 minutes in the ambulatory care pharmacy clinics, adjacent to the physicians’ clinics, where pharmacists review patients’ medications, dosages, indications, and provide patient counseling. In addition, patients ‘medical records were reviewed to capture any documented side effects of those who consented to be interviewed, and the data were entered in Microsoft Excel spreadsheet and coded for analysis.

### 2.3. Ethical Considerations

This research was approved by the institutional review board of the College of Medicine at King Saud University (Approval number: E-21-6071). All participants had signed a written consent form and the study adhered to the ethical principles of the Helsinki Declaration. 

### 2.4. Data Analysis

The minimum sample size needed for this study was estimated to be 220 participants based on α = 0.05, β = 0.05, power of 95%, and medium effect size (e.g., Cohen’s w = 0.3). Descriptive statistics using frequencies, percentages, means, and standard deviation (SD) were performed for demographic characteristics, biologic drug use, type of vaccination, and the reported vaccine side effects. Chi-square and Fisher’s exact tests were performed as appropriate to compare the percentages of COVID-19 vaccine side effects between those who received one and two doses. The relationships between the indications of the biological products (e.g., IBD, psoriasis, rheumatoid arthritis) and the COVID-19 vaccine side effects were examined using Pearson’s correlation coefficient (r). Univariate logistic regressions were conducted to examine associations between different commonly reported side effects (e.g., fever, fatigue, headache, muscle pain, hypotension, diarrhea, loss of smell or taste) that have been reported by ≥5% of participants and the vaccine types (Oxford–AstraZeneca and Pfizer–BioNTech). Additionally, the individual relationships between age, gender, number of vaccine doses, and the commonly reported side effects were examined using univariate logistic regressions since differences in the rates of reported COVID-19 adverse events were reported across gender and age and number of vaccine doses [35,36,37]. All statistical analyses were conducted using SAS^®^ version 9.4 (SAS^®^ Institute, Cary, NC, USA).

## 3. Results

Between August 2021 and December 2021, around 600 patients were contacted. Of those, 417 patients with no previous history of COVID-19 infection consented to participate and were interviewed (280 patients from Riyadh and 137 from Tabuk). None of the participants had any allergy to vaccines, their mean age was 38.5 years, and ~53% of them were females. Regarding medical conditions, about 35% of the participants had IBD, followed by rheumatoid arthritis (RA) (26.14%). Nearly half of the participants (46.51%) were treated with anti-TNFs, such as infliximab and adalimumab. The most commonly encountered comorbid medical conditions were hypertension (*n* = 68, 16.31%), diabetes (*n* = 49, 11.75%), and dyslipidemia (*n* = 40, 9.59%). More than two-thirds of the participants were either overweight or obese (*n* = 305, 73.14%), around 60% had a college degree or higher, and a similar proportion were married. Most of the participants received Pfizer–BioNTech vaccine (82%), and the majority (70.98%) received two doses of the vaccine as shown in Table 1.

**Figure 1:**
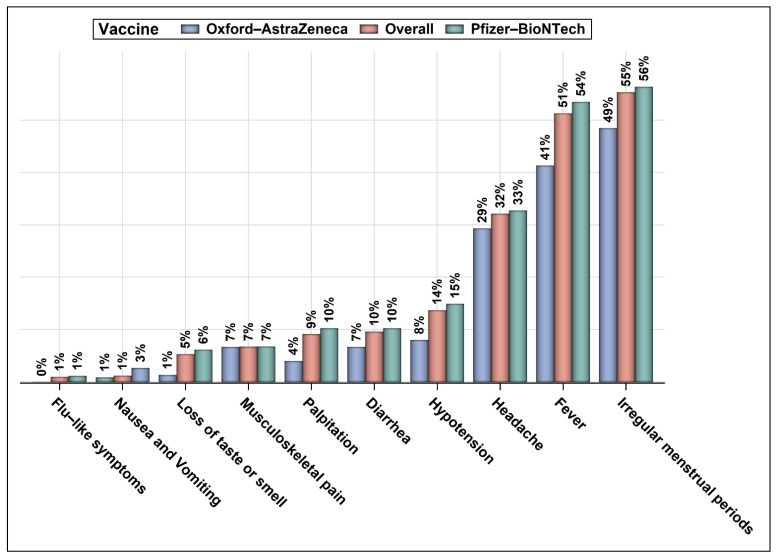
Rates of commonly reported side effects of COVID-19 vaccines.

Fever, headache, and palpitation were the most commonly reported side effects of both Oxford–AstraZeneca and Pfizer–BioNTech vaccines, as shown in Figure 1. Although the rates of most reported side effects were higher among patients who received Pfizer–BioNTech vaccine, the difference was not statistically significant. Moreover, no difference in the rates of fever, headache, palpitation, hypotension, flu-like symptoms, musculoskeletal pain, nausea and vomiting, loss of taste or smell, and diarrhea was found between those who received one dose and two doses of COVID-19 vaccine, as shown in Table 2. However, a higher percentage of female patients reported menstrual irregularities, such as changes in the cycle length and unexpected vaginal bleeding before or after the expected periods, after the first dose of COVID-19 vaccine in comparison to those who received two doses of the vaccine (67.19% vs. 50.33%, *p* = 0.023). Table 3 shows the Pearson’s correlation coefficients (r) of different commonly reported COVID-19 side effects with different indications for the biological therapies. The reporting of fever as a side effect of COVID-19 vaccines was positively correlated with certain autoimmune disorders, such as multiple sclerosis (MS) and dermatological disorders (e.g., psoriasis). Palpitation was negatively associated with MS and inflammatory bowel disease (IBD) and positively associated with rheumatoid arthritis (RA). Hypotension was positively associated with dyslipidemia and negatively associated with IBD. Menstrual irregularities were positively associated with RA and negatively associated with MS and IBD. Moreover, headache was positively associated with MS and dermatological disorders and negatively associated with asthma. Interestingly, loss of smell or taste was positively associated with asthma. 

Approximately 50% of the female participants reported transient irregular menstrual periods post-vaccination with no significant difference between the two vaccines (Oxford–AstraZeneca, Pfizer–BioNTech). However, higher odds of reporting palpitation (OR = 1.033, 95% CI [1.009–1.058], *p* = 0.0063) and hypotension (OR = 1.023, 95% CI [1.003–1.044], *p* = 0.0226) post-vaccination were associated with older age (e.g., the odds of palpitation and hypotension were higher by 3.3% and 2.3%, respectively, for each additional year of age). Male participants had lower odds of reporting palpitation post-vaccination in comparison to their female counterparts (OR = 0.254, 95% CI [0.114–0.569], *p* = 0.0009). Furthermore, older age was associated with higher odds of reporting diarrhea post-vaccination (OR = 1.034, 95% CI [1.011–1.058], *p* = 0.0043), while males had lower odds of reporting diarrhea in comparison to their female counterparts (OR = 0.321, 95% CI [0.153–0.676], *p* = 0.0028). Moreover, the older the age of females, the lower their odds of reporting irregular menstrual periods (OR = 0.923, 95% CI [0.900–0.947], *p* < 0.0001). Likewise, those who received the second dose of the vaccine reported lower odds of irregular menstrual periods (OR = 0.495, 95% CI [0.268–0.912], *p* = 0.0242), as shown in Table 4.

## 4. Discussion

In this descriptive study, we explored the types and rates of COVID-19 vaccines administered to patients with different autoimmune disorders as well as dyslipidemia and asthma on biologics. Only two COVID-19 vaccines (Oxford–AstraZeneca, Pfizer–BioNTech) were administered, and the majority (82%) received Pfizer–BioNTech vaccine, although six COVID-19 vaccines have been approved by the Saudi Food and Drug Authority (SFDA) [38]. The COVID-19 vaccine side effects are predictable since they are related to the innate immune system, whereby a flurry of white blood cells arrives at the site of injection right after the administration of the vaccine, resulting in the production of cytokines, which trigger a broad range of symptoms, such as fever, chills, and fatigue [39]. In this study, fever, headache, hypotension, diarrhea, palpitation and musculoskeletal pain were the most commonly reported vaccine side effects. These side effects are similar to the those reported by members of the general population in Saudi Arabia who received the same vaccines [20,21,40]. However, the rates of these side effects were much lower in comparison to the ones reported in prior studies [20,21,40]. For example, the percentage of participants who reported having fever post-vaccination was approximately 51% in comparison to 66% and 73% in two other studies that explored the rates of common vaccine side effects among the general population in Saudi Arabia [21,40]. This could potentially be related to the weakened humoral responses to vaccines among patients with autoimmune diseases and those on biologic therapies [25,29,41]. Although no statistically significant difference in the rates of reported side effects between the two used vaccines (Oxford–AstraZeneca, Pfizer–BioNTech) was found, patients who received Pfizer–BioNTech had higher rates of diarrhea, loss of smell or appetite, palpitation, hypotension, fever, and irregular menstrual periods among females. This is at odds with most published studies that explored vaccine adverse events among the general population as well as those with autoimmune diseases and found that the rates of side effects for Oxford–AstraZeneca vaccine were overall higher than the rates for mRNA-based vaccines such as Moderna and Pfizer–BioNTech [21,34]. Another interesting side effect that was reported by more than 50% of the female participants was irregular menstrual periods following vaccination. Although this side effect was not commonly reported in several studies that explored the rates and types of COVID-19 side effects [14,20,21,33,34], it was listed in several vaccine safety reports [42]. This finding is in contrast to the findings from a recently published prospective study that tracked a cohort of 2403 vaccinated women aged 18 to 45 years in the United States to examine the impact of COVID-19 vaccination on their menstrual cycles and compare that to another cohort of 1556 unvaccinated women from a similar age group. In that study, only a small and non-significant change in the menstrual cycle’s length was found among the vaccinated women [43]. This could be attributable to the differences in the underlying diseases and sociodemographic characteristics of the patients in our study. Additionally, some side effects were associated more with certain diseases, such as the loss of smell or taste with asthma or headache with MS. These associations between particular COVID-19 vaccine side effects and certain diseases that different biological therapies are indicated for should be investigated further to explain whether the reported side effects are caused by the COVID-19 vaccines or are just symptoms of these diseases.

Palpitation and hypotension, which can be symptoms of rare side effects such as myocarditis and myopericarditis, have been more frequently reported by participants who were vaccinated with the Pfizer–BioNTech vaccine or documented in their medical charts following vaccination. Myocarditis and myopericarditis were reported more often for patients, especially females, who received mRNA vaccines, such as Pfizer–BioNTech vaccine [17]. However, there was no confirmation that these side effects were related to myocarditis or myopericarditis. The older the patients, the higher the incidence of reporting or having hypotension and/or palpitation as well as diarrhea post-vaccination, which is at odds with another questionnaire-based study that explored the rates of COVID-19 side effects among the Saudi general population using different social media platforms and that found a lower incidence of fast heartbeat or palpitation among older adults (≥60 years) [44]. However, in another study that examined the rates of serious adverse events related to COVID-19 vaccines across age and gender groups using the Vaccine Adverse Event Reporting System (VAERS) database in the United States, the rates of serious adverse events, such as permanent disability, hospitalization, and death were significantly higher among older adults (≥65 years) in comparison to their younger counterparts [35]. Furthermore, among females, older age was associated with lower odds of having irregular menstrual periods. Moreover, females had higher odds of reporting diarrhea post-vaccination. This is consistent with most studies that reported higher rates of COVID-19 vaccine side effects among females in comparison to their male counterparts across all age groups [36]. Nonetheless, other studies have reported higher rates of serious adverse events, such as hospitalization and death, after COVID-19 vaccination among males [35]. Although second or third doses of COVID-19 vaccines were associated with higher rates of adverse events, [45,46] this relationship between the number of COVID-19 vaccine doses and different side effects was not found in this study. However, the rate of menstrual irregularities was higher among females after the first dose of the vaccine in comparison to those who received two doses.

Although to the best of our knowledge this is the first study that explored the side effects of two commonly used vaccines (Oxford–AstraZeneca and Pfizer–BioNTech) in Saudi Arabia, it has several limitations. First, recall bias cannot be ruled out since patients were asked about the side effects they experienced post-vaccination, which could have been several weeks or months prior to being interviewed for this study. Secondly, interviewer bias cannot be ruled out, either, since different interviewers were involved in the data collection in three tertiary care centers, although a structured interview protocol was believed to have been followed by all three interviewers. Thirdly, although the study included patients in three different tertiary care centers in Saudi Arabia, the study findings cannot be generalized to other patients on mAbs who are seen in other hospitals in different regions in Saudi Arabia. Additionally, the questionnaire’s reliability was not examined, mainly because the study aimed to explore and describe the rates of different COVID-19 vaccine side effects among patients on mAbs.

## 5. Conclusions

COVID-19 vaccines appear to be largely safe with no serious side effects among patients treated with mAbs. Patients with autoimmune diseases and those treated with mAbs should be encouraged to get vaccinated as soon as possible due to their higher likelihood of viral infections, and COVID-19 is not an exception. Future studies with more representative samples and more robust study designs should be conducted to explore the types, rates, and severity of different COVID-19 vaccine side effects among immunocompromised patients, such as patients with autoimmune diseases and those treated with biological therapies, such as mAbs. In addition, the impact of COVID-19 vaccines on the patients’ underlying autoimmune diseases should be examined in future research.

## Figures and Tables

**Table 1:** Demographic and medical characteristics of participants.

Characteristics	Patients
	*n* = 417
Age (Years), Mean ± (SD)	38.5 ± 13.57
Gender	
Female (%)	215 (51.56)
Male (%)	202 (48.44)
Body mass index (BMI)	
Underweight ≤ 18.5 (%)	17 (4.08)
Normal weight = 18.5–24.9 (%)	95 (22.78)
Overweight = 25–29.9 (%)	136 (32.61)
Obesity = BMI of 30 or greater (%)	169 (40.53)
Educational level	
No formal education (%)	8 (2.86)
Elementary school (%)	20 (4.80)
Intermediate school (%)	23 (5.52)
Secondary school (%)	116 (27.82)
College or Associate degree (%)	224 (53.72)
Postgraduate degree (%)	26 (6.24)
Marital status	
Single (%)	135 (32.37)
Married (%)	253 (60.67)
Widowed (%)	12 (2.88)
Divorced (%)	17 (4.08)
Employment status	
Employed (%)	250 (59.95)
Unemployed (%)	167 (40.05)
Smoker	
Yes (%)	108 (25.9)
No (%)	309 (74.10)
Main Disease	
Dermatological disorders (e.g., Psoriasis) (%)	57 (13.67)
Inflammatory bowel disease (IBD) (%)	147 (35.25)
Multiple sclerosis (MS) (%)	40 (9.59)
Rheumatoid arthritis (RA) (%)	109 (26.14)
Asthma (%)	14 (3.36)
Dyslipidemia (%)	18 (4.32)
Others ⊥ (%)	32 (7.68)
Biological medications	
Abatacept (%)	6 (1.44)
Adalimumab (%)	92 (22.06)
Infliximab (%)	102 (24.46)
Natalizumab (%)	28 (6.71)
Ocrelizumab (%)	3 (0.72)
Rituximab (%)	18 (4.32)
Risankizumab (%)	20 (7.14)
Tocilizumab (%)	29 (6.95)
Vedolizumab (%)	19 (4.56)
Belimumab (%)	3 (0.72)
Certolizumab (%)	12 (2.88)
Etanercept (%)	21 (5.04)
Ustekinumab (%)	24 (5.76)
Mepolizumab (%)	18 (4.32)
Dupilumab (%)	10 (2.40)
Evolocumab (%)	2 (0.48)
Omalizumab (%)	10 (2.40)
Comorbid conditions	
Hypertension (%)	68 (16.31)
Diabetes mellitus (%)	49 (11.75)
Dyslipidemia (%)	40 (9.59)
Renal disorders (%)	5 (1.20)
Type of vaccine	
Oxford–AstraZeneca (%)	(17.99)75
Pfizer–BioNTech (%)	342 (82.01)
Number of vaccine doses	
Partially immunized (first dose)	121(29.02)
Fully immunized (second dose)	296(70.98)

⊥ Adult onset Still’s disease (AOSD), ankylosing spondylitis, Behçet’ s disease (BD), giant cell arteritis (GCA), hidradenitis suppurativa (HS), myositis, neuromyelitis optica spectrum disorder (NMOSD), relapsing polychondritis, fibromyalgia, systemic lupus erythematosus (SLE), myasthenia gravis and Sjogren’s syndrome.

**Table 2:** The frequency of commonly reported COVID-19 vaccine side effects based on the number of doses.

Side Effect	First Dose *n* (%)	Second Dose *n* (%)	*p*-Value
Flu-like symptoms	1 (0.83)	3 (1.01)	0.858
Nausea and vomiting	3 (2.48)	2 (0.68)	0.1246
Loss of taste or smell	9 (7.44)	13 (4.39)	0.229
Musculoskeletal pain	9 (7.44)	19 (6.42)	0.705
Palpitation	12 (9.92)	26 (8.78)	0.715
Diarrhea	11 (9.09)	29 (9.80)	0.824
Hypotension	20 (16.53)	37 (12.50)	0.277
Headache	33 (27.27)	101 (34.12)	0.174
Fever	68 (56.20)	146 (49.32)	0.202
Irregular menstrual periods among females	43 (67.19)	76 (50.33)	0.0230 *

* *p*-value < 0.05.

**Table 3:** The Pearson’s correlation coefficients (r) between different indications for the biological therapies and commonly reported COVID-19 vaccine side effects.

Autoimmune Disease	Fever	Flu-like Symptoms	Musculoskeletal Pain	Nausea and Vomiting	Loss of Taste or Smell	Palpitation	Hypotension	Irregular Menstrual Periods	Headache
MS	0.10545 *	0.0515	0.043	−0.035	−0.07687	−0.103 *	0.012	−0.208 *	0.142 *
IBD	−0.095	−0.016	0.138 *	0.063	0.017	−0.186 *	−0.204 *	−0.212 *	−0.011
Dermatological disorders	0.118 *	−0.028	−0.041	−0.031	−0.068	−0.028	−0.088	0.059	0.110 *
RA	0.001	0.053	−0.094	0.034	−0.067	0.171 *	0.065	0.279 *	−0.047
Asthma	−0.004	−0.018	−0.050	−0.020	0.194 *	0.079	0.080	−0.023	−0.128 *
Dyslipidemia	−0.005	−0.020	−0.056	−0.023	−0.050	0.055	0.293 *	−0.107	−0.019

Abbreviation: MS = multiple sclerosis, IBD = inflammatory bowel disease, RA = rheumatoid arthritis. * *p*-value < 0.05.

**Table 4:** Univariate logistic regressions for the association between the most commonly reported vaccine side effects (fever, headache, palpitation, hypotension, diarrhea, loss of smell or taste, musculoskeletal pain, and irregular menstrual periods) and vaccine type, age, gender, and number of vaccine doses.

Fever				
Variable	Odds Ratio (OR)	95% Confidence Interval (CI)		*p*-Value
Pfizer–BioNTech vs. Oxford–AstraZeneca	1.634	0.985	2.710	0.0575
Age	1.003	0.989	1.018	0.658
Male vs. Female	0.903	0.615	1.326	0.6016
Second dose vs. First dose	1.318	0.862	2.017	0.2030
Headache				
**Variable**	**Odds Ratio (OR)**	**95% Confidence Interval (CI)**		***p*** ***-*** **Value**
Pfizer–BioNTech vs. Oxford–AstraZeneca	1.173	0.680	2.025	0.5665
Age	1.003	0.988	1.018	0.7421
Male vs. Female	1.049	0.695	1.583	0.8193
Second dose vs. First dose	0.724	0.454	1.155	0.1750
Palpitation				
**Variable**	**Odds Ratio (OR)**	**95% Confidence Interval (CI)**		***p*** ***-*** **Value**
Pfizer–BioNTech vs. Oxford–AstraZeneca	2.735	0.818	9.140	0.1021
Age	1.033	1.009	1.058	0.0063 *
Male vs. Female	0.254	0.114	0.569	0.0009 *
Second dose vs. First dose	1.143	0.557	2.347	0.7152
Hypotension				
**Variable**	**Odds Ratio (OR)**	**95% Confidence Interval (CI)**		***p*** ***-*** **Value**
Pfizer–BioNTech vs. Oxford–AstraZeneca	2.015	0.831	4.887	0.1209
Age	1.023	1.003	1.044	0.0226 *
Male vs. Female	0.808	0.460	1.418	0.4569
Second dose vs. First dose	1.386	0.768	2.502	0.2783
Diarrhea				
**Variable**	**Odds Ratio (OR)**	**95% Confidence Interval (CI)**		***p*** ***-*** **Value**
Pfizer–BioNTech vs. Oxford–AstraZeneca	1.596	0.604	4.220	0.3460
Age	1.034	1.011	1.058	0.0043 *
Male vs. Female	0.321	0.153	0.676	0.0028 *
Second dose vs. First dose	0.921	0.444	1.908	0.8241
Loss of smell or taste				
**Variable**	**Odds Ratio (OR)**	**95% Confidence Interval (CI)**		***p*** ***-*** **Value**
Pfizer–BioNTech vs. Oxford–AstraZeneca	4.841	0.641	36.561	0.1263
Age	0.986	0.953	1.019	0.3959
Male vs. Female	1.574	0.658	3.767	0.3080
Second dose vs. First dose	1.749	0.727	4.207	0.2117
Musculoskeletal pain				
**Variable**	**Odds Ratio (OR)**	**95% Confidence Interval (CI)**		***p*** ***-*** **Value**
Pfizer–BioNTech vs. Oxford–AstraZeneca	1.009	1.009	2.747	0.9854
Age	0.982	0.952	1.012	0.2380
Male vs. Female	0.917	0.425	1.978	0.8254
Second dose vs. First dose	1.172	0.514	2.668	0.7061
Irregular menstrual periods among females				
**Variable**	**Odds Ratio (OR)**	**95% Confidence Interval (CI)**		***p*** ***-*** **Value**
Pfizer–BioNTech vs. Oxford–AstraZeneca	0.718	0.320	1.612	0.4223
Age	0.923	0.900	0.947	<0.0001 *
Second dose vs. First dose	0.495	0.268	0.912	0.0242 *

* *p* < 0.05.

## Data Availability

The data are available upon request to the corresponding author (Y.A.).

## Appendix A

1. Gender
  ○ Male
  ○ Female
2. Age: __________
3. City: __________
4. Hospital: _____________
5. Body Mass Index (BMI):
  ○ Underweight (≤18.5)
  ○ Normal weight (18.5–24.9)
  ○ Overweight (25–29.9)
  ○ Obesity (≥30)
6. Marital Status:
  ○ Single
  ○ Married
  ○ Divorced
  ○ Widowed
7. Educational Level:
  ○ No formal education
  ○ Elementary school
  ○ Intermediate school
  ○ Secondary school
  ○ College or associate degree
  ○ Postgraduate degree
8. Employment Status:
  ○ Employed
  ○ Unemployed
9. Smoking Status:
  ○ Non–smoker.
  ○ Smoker.
10. The indication that biological therapy is prescribed for:
  ○ Inflammatory Bowel Disease (IBD)
  ○ Multiple Sclerosis (MS)
  ○ Rheumatoid arthritis (RA)
  ○ Psoriasis
  ○ Familial Hypercholesterolemia.
  ○ Asthma
  ○ Other: _____________
11. Other Medical Conditions
  ○ Diabetes
  ○ Hypertension
  ○ Asthma/COPD
  ○ Dyslipidemia
  ○ Other: ___________________
12. Biological Therapy:
  ○ Infliximab
  ○ Natalizumab
  ○ Vedolizumab
  ○ Belimumab
  ○ Ustekinumab
  ○ Rituximab
  ○ Tocilizumab
  ○ Other: ________________
13. Date of the last biological therapy administration: ____________
14. Other Prescription Medications: _____________________________
15. Vaccinated against COVID–19:
  ○ Yes
  ○ No
16. Vaccinated 1st dose
  ○ Date of Vaccination: _______________
17. Type of Vaccine:
  ○ Pfizer–BioNTech
  ○ Oxford–AstraZeneca
18. Vaccinated 2nd dose
  ○ Date of Vaccination: _______________
19. Type of Vaccine:
  ○ Pfizer–BioNTech
  ○ Oxford–AstraZeneca
20. Any side effects from the vaccine:
  ○ Tiredness
  ○ Headache
  ○ Muscle pain
  ○ Chills
  ○ Diarrhea
  ○ Abdominal pain
  ○ Constipation
  ○ Oral hypoesthesia
  ○ Mouth ulceration
  ○ Lip/tongue swelling
  ○ Fever
  ○ Nausea
  ○ Fainting/hypotension
  ○ Fever
  ○ Severe allergic reactions
  ○ Bleeding
  ○ Coagulopathy disorders
  ○ Palpitations
  ○ Ear Pain
  ○ Eye Pain/photophobia/blurred vision
  ○ Injection site pain
  ○ Influenza-like symptoms
  ○ Other: ________________________
21. Any previous allergy to vaccines?
  ○ Yes
  ○ No
22. Have you been infected by COVID-19 before vaccination?
  ○ Yes
  ○ No
23. Have you been infected by COVID-19 after vaccination?
  ○ Yes
  ○ No
24. Did you experience any worsening of your disease symptoms after vaccination?
  ○ Yes
  ○ No
25. Have you been hospitalized after vaccination?
  ○ Yes
  ○ No
26. Did you visit any public or private clinic because of the vaccine’s side effects?
  ○ Yes
  ○ No

## References

[1] Cucinotta D., Vanelli M. (2020). WHO declares COVID-19 a pandemic. Acta Biomed..

[2] Viner R.M., Ward J.L., Hudson L.D., Ashe M., Patel S.V., Hargreaves D., Whittaker E. (2020). Systematic review of reviews of symptoms and signs of COVID-19 in children and adolescents. Arch. Dis. Child..

[3] Worl Health Organization WHO Coronavirus (COVID-19) Dashboard. Worl Health Organization. https://covid19.who.int/.

[4] Ministry of Health Saudi Ministry of Health COVID-19 Dashboard. https://covid19.moh.gov.sa/.

[5] AlRuthia Y., Al-Salloum H.F., Almohammed O.A., Alqahtani A.S., Al-Abdulkarim H.A., Alsofayan Y.M., Almudarra S.S., AlQahtani S.H., Almutlaq A., Alabdulkareem K. (2022). Demographic Characteristics and Status of Vaccinated Individuals with a History of COVID-19 Infection Pre- or Post-Vaccination: A Descriptive Study of a Nationally Representative Sample in Saudi Arabia. Vaccines.

[6] Fiolet T., Kherabi Y., MacDonald C.J., Ghosn J., Peiffer-Smadja N. (2022). Comparing COVID-19 vaccines for their characteristics, efficacy and effectiveness against SARS-CoV-2 and variants of concern: A narrative review. Clin. Microbiol. Infect..

[7] Ndwandwe D., Wiysonge C.S. (2021). COVID-19 vaccines. Curr. Opin. Immunol..

[8] Self W.H., Tenforde M.W., Rhoads J.P., Gaglani M., Ginde A.A., Douin D.J., Olson S.M., Talbot H.K., Casey J.D., Mohr N.M. (2021). Comparative Effectiveness of Moderna, Pfizer-BioNTech, and Janssen (Johnson & Johnson) Vaccines in Preventing COVID-19 Hospitalizations among Adults without Immunocompromising Conditions.

[9] Voysey M., Clemens S.A.C., Madhi S.A., Weckx L.Y., Folegatti P.M., Aley P.K., Angus B., Baillie V.L., Barnabas S.L., Bhorat Q.E. (2021). Safety and efficacy of the ChAdOx1 nCoV-19 vaccine (AZD1222) against SARS-CoV-2: An interim analysis of four randomised controlled trials in Brazil, South Africa, and the UK. Lancet.

[10] Mahase E. (2021). COVID-19: Novavax vaccine efficacy is 86% against UK variant and 60% against South African variant. BMJ.

[11] Ranzani O.T., Hitchings M.D.T., Dorion M., D’Agostini T.L., de Paula R.C., de Paula O.F.P., Villela E.F.D.M., Torres M.S.S., de Oliveira S.B., Schulz W. (2021). Effectiveness of the CoronaVac vaccine in older adults during a gamma variant associated epidemic of COVID-19 in Brazil: Test negative case-control study. BMJ.

[12] Tregoning J.S., Flight K.E., Higham S.L., Wang Z., Pierce B.F. (2021). Progress of the COVID-19 vaccine effort: Viruses, vaccines and variants versus efficacy, effectiveness and escape. Nat. Rev. Immunol..

[13] Bin Lee A.R.Y., Wong S.Y., Chai L.Y.A., Lee S.C., Lee M.X., Muthiah M.D., Tay S.H., Teo C.B., Tan B.K.J., Chan Y.H. (2022). Efficacy of COVID-19 vaccines in immunocompromised patients: Systematic review and meta-analysis. BMJ.

[14] Centers for Disease Control and Prevention (2021). Selected Adverse Events Reported after COVID-19 Vaccination. Google Scholar. https://www.cdc.gov/coronavirus/2019-ncov/vaccines/safety/adverse-events.html.

[15] Shavit R., Maoz-Segal R., Iancovici-Kidon M., Offengenden I., Yahia S.H., Maayan D.M., Lifshitz-Tunitsky Y., Niznik S., Frizinsky S., Deutch M. (2021). Prevalence of allergic reactions after Pfizer-BioNTech COVID-19 vaccination among adults with high allergy risk. JAMA Netw. Open.

[16] Pottegård A., Lund L.C., Karlstad Ø., Dahl J., Andersen M., Hallas J., Lidegaard Ø., Tapia G., Gulseth H.L., Ruiz P.L.D. (2021). Arterial events, venous thromboembolism, thrombocytopenia, and bleeding after vaccination with Oxford-AstraZeneca ChAdOx1-S in Denmark and Norway: Population based cohort study. BMJ.

[17] Husby A., Hansen J.V., Fosbøl E., Thiesson E.M., Madsen M., Thomsen R.W., Sørensen H.T., Andersen M., Wohlfahrt J., Gislason G. (2021). SARS-CoV-2 vaccination and myocarditis or myopericarditis: Population based cohort study. BMJ.

[18] Woo E.J., Mba-Jonas A., Dimova R.B., Alimchandani M., Zinderman C.E., Nair N. (2021). Association of Receipt of the Ad26.COV2.S COVID-19 Vaccine With Presumptive Guillain-Barré Syndrome, February–July 2021. JAMA.

[19] Oh H.-K., Kim E.K., Hwang I., Kim T.E., Lee Y.-K., Lee E. (2021). COVID-19 vaccine safety monitoring in the Republic of Korea: 26 February 2021 to 30 April 2021. Osong Public Health Res. Perspect..

[20] Alghamdi A.A., Alkazemi A., Alissa A., Alghamdi I., Alwarafi G., Waggas H.A. (2021). Adverse Events following AstraZeneca COVID-19 Vaccine in Saudi Arabia: A Cross-Sectional Study among Healthcare and Nonhealthcare Workers. Intervirology.

[21] Alhazmi A., Alamer E., Daws D., Hakami M., Darraj M., Abdelwahab S., Maghfuri A., Algaissi A. (2021). Evaluation of Side Effects Associated with COVID-19 Vaccines in Saudi Arabia. Vaccines.

[22] Riad A., Pokorná A., Attia S., Klugarová J., Koščík M., Klugar M. (2021). Prevalence of COVID-19 Vaccine Side Effects among Healthcare Workers in the Czech Republic. J. Clin. Med..

[23] Melmed G.Y., Ippoliti A.F., Papadakis K.A., Tran T.T., Birt J.L., Lee S.K., Frenck R.W., Targan S.R., Vasiliauskas E.A. (2006). Patients with Inflammatory Bowel Disease Are at Risk for Vaccine-Preventable Illnesses. Am. J. Gastroenterol..

[24] Subesinghe S., Bechman K., Rutherford A.I., Goldblatt D., Galloway J.B. (2018). A Systematic Review and Metaanalysis of Antirheumatic Drugs and Vaccine Immunogenicity in Rheumatoid Arthritis. J. Rheumatol..

[25] Ciotti J.R., Valtcheva M.V., Cross A.H. (2020). Effects of MS disease-modifying therapies on responses to vaccinations: A review. Mult. Scler. Relat. Disord..

[26] Arnold J., Winthrop K., Emery P. (2021). COVID-19 vaccination and antirheumatic therapy. Rheumatology.

[27] Van Assen S., Holvast A., Benne C.A., Posthumus M.D., Van Leeuwen M.A., Voskuyl A.E., Blom M., Risselada A.P., de Haan A., Westra J. (2010). Humoral responses after influenza vaccination are severely reduced in patients with rheumatoid arthritis treated with rituximab. Arthritis Rheum..

[28] Arad U., Tzadok S., Amir S., Mandelboim M., Mendelson E., Wigler I., Sarbagil-Maman H., Paran D., Caspi D., Elkayam O. (2011). The cellular immune response to influenza vaccination is preserved in rheumatoid arthritis patients treated with rituximab. Vaccine.

[29] deBruyn J., Fonseca K., Ghosh S., Panaccione R., Gasia M.F., Ueno A., Kaplan G.G., Seow C.H., Wrobel I. (2016). Immunogenicity of influenza vaccine for patients with inflammatory bowel disease on maintenance infliximab therapy: A randomized trial. Inflamm. Bowel Dis..

[30] Gabay C., Bel M., Combescure C., Ribi C., Meier S., Posfay-Barbe K., Grillet S., Seebach J.D., Kaiser L., Wunderli W. (2011). Impact of synthetic and biologic disease-modifying antirheumatic drugs on antibody responses to the AS03-adjuvanted pandemic influenza vaccine: A prospective, open-label, parallel-cohort, single-center study. Arthritis Rheum..

[31] Tang W., Askanase A.D., Khalili L., Merrill J.T. (2021). SARS-CoV-2 vaccines in patients with SLE. Lupus Sci. Med..

[32] Bijlsma J.W. (2021). EULAR December 2020 viewpoints on SARS-CoV-2 vaccination in patients with RMDs. Ann. Rheum. Dis..

[33] Al Khames Aga Q.A., Alkhaffaf W.H., Hatem T.H., Nassir K.F., Batineh Y., Dahham A.T., Shaban D., Aga L.A., Agha M.Y.R., Traqchi M. (2021). Safety of COVID-19 vaccines. J. Med. Virol..

[34] Esquivel-Valerio J.A., Skinner-Taylor C.M., Moreno-Arquieta I.A., la Garza J.A.C.-D., Garcia-Arellano G., Gonzalez-Garcia P.L., Almaraz-Juarez F.d.R., Galarza-Delgado D.A. (2021). Adverse events of six COVID-19 vaccines in patients with autoimmune rheumatic diseases: A cross-sectional study. Rheumatol. Int..

[35] Xiong X., Yuan J., Li M., Jiang B., Lu Z.K. (2021). Age and Gender Disparities in Adverse Events Following COVID-19 Vaccination: Real-World Evidence Based on Big Data for Risk Management. Front. Med..

[36] Green M.S., Peer V., Magid A., Hagani N., Anis E., Nitzan D. (2022). Gender Differences in Adverse Events Following the Pfizer-BioNTech COVID-19 Vaccine. Vaccines.

[37] Beatty A.L., Peyser N.D., Butcher X.E., Cocohoba J.M., Lin F., Olgin J.E., Pletcher M.J., Marcus G.M. (2021). Analysis of COVID-19 Vaccine Type and Adverse Effects Following Vaccination. JAMA Netw. Open.

[38] Gazette S. (2021). MoH: Six COVID-19 Vaccines Approved in Saudi Arabia.

[39] Chapin-Bardales J., Gee J., Myers T. (2021). Reactogenicity Following Receipt of mRNA-Based COVID-19 Vaccines. JAMA.

[40] Darraj M.A., Al-Mekhlafi H.M. (2022). Prospective Evaluation of Side-Effects Following the First Dose of Oxford/AstraZeneca COVID-19 Vaccine among Healthcare Workers in Saudi Arabia. Vaccines.

[41] Efrati S., Catalogna M., Abu Hamad R., Hadanny A., Bar-Chaim A., Benveniste-Levkovitz P., Levtzion-Korach O. (2021). Safety and humoral responses to BNT162b2 mRNA vaccination of SARS-CoV-2 previously infected and naive populations. Sci. Rep..

[42] Male V. (2021). Menstrual changes after COVID-19 vaccination. BMJ.

[43] Edelman A., Boniface E.R., Benhar E., Han L., Matteson K.A., Favaro C. (2022). Association Between Menstrual Cycle Length and Coronavirus Disease 2019 (COVID-19) Vaccination: A U.S. Cohort. Obstet. Gynecol..

[44] El-Shitany N.A., Harakeh S., Badr-Eldin S.M., Bagher A.M., Eid B., Almukadi H. (2021). Minor to Moderate Side Effects of Pfizer-BioNTech COVID-19 Vaccine Among Saudi Residents: A Retrospective Cross-Sectional Study. Int. J. Gen. Med..

[45] Alamer E., Alhazmi A., Qasir N.A., Alamer R., Areeshi H., Gohal G. (2021). Side Effects of COVID-19 Pfizer-BioNTech mRNA Vaccine in Children Aged 12–18 Years in Saudi Arabia. Vaccines.

[46] Andrzejczak-Grządko S., Czudy Z., Donderska M. (2021). Side effects after COVID-19 vaccinations among residents of Poland. Eur. Rev. Med. Pharmacol. Sci..

